# New Insights on NETosis Induced by *Entamoeba histolytica*: Dependence on ROS from Amoebas and Extracellular MPO Activity

**DOI:** 10.3390/antiox10060974

**Published:** 2021-06-18

**Authors:** César Díaz-Godínez, Joshue Fabián Jorge-Rosas, Mario Néquiz, Santiago Martínez-Calvillo, Juan P. Laclette, Carlos Rosales, Julio C. Carrero

**Affiliations:** 1 Departamento de Inmunología, Instituto de Investigaciones Biomédicas, Universidad Nacional Autónoma de México (UNAM), Ciudad de México 04510, Mexico; cesar_rha32@hotmail.com (C.D.-G.); a.fabian.sfrayer@gmail.com (J.F.J.-R.); laclette@biomedicas.unam.mx (J.P.L.); 2 Unidad de Investigación en Medicina Experimental, Facultad de Medicina, Universidad Nacional Autónoma de México, Ciudad de México 06720, Mexico; manequiz@yahoo.com.mx; 3 Unidad de Biomedicina, Facultad de Estudios Superiores Iztacala, Universidad Nacional Autónoma de México, Av. de los Barrios 1, Col. Los Reyes Iztacala, Tlalnepantla, Estado de México 54090, Mexico; scalv@unam.mx

**Keywords:** *Entamoeba histolytica*, neutrophil extracellular traps (NETs), neutrophils, reactive oxygen species (ROS), myeloperoxidase

## Abstract

NETosis is a neutrophil process involving sequential steps from pathogen detection to the release of DNA harboring antimicrobial proteins, including the central generation of NADPH oxidase dependent or independent ROS. Previously, we reported that NETosis triggered by *Entamoeba histolytica* trophozoites is independent of NADPH oxidase activity in neutrophils, but dependent on the viability of the parasites and no ROS source was identified. Here, we explored the possibility that *E. histolytica* trophozoites serve as the ROS source for NETosis. NET quantitation was performed using SYTOX^®^ Green assay in the presence of selective inhibitors and scavengers. We observed that respiratory burst in neutrophils was inhibited by trophozoites in a dose dependent manner. Mitochondrial ROS was not also necessary, as the mitochondrial scavenger mitoTEMPO did not affect the process. Surprisingly, ROS-deficient amoebas obtained by pre-treatment with pyrocatechol were less likely to induce NETs. Additionally, we detected the presence of MPO on the cell surface of trophozoites after the interaction with neutrophils and found that luminol and isoluminol, intracellular and extracellular scavengers for MPO derived ROS reduced the amount of NET triggered by amoebas. These data suggest that ROS generated by trophozoites and processed by the extracellular MPO during the contact with neutrophils are required for *E. histolytica* induced NETosis.

## 1. Introduction

Neutrophil extracellular traps (NETs) are DNA fibers associated with histones and antimicrobial proteins that are released by neutrophils into the extracellular space in a process denominated NETosis [1]. Initially, NETs were described as a strategy used for neutrophils to entrap and kill microorganisms [2,3,4,5]; nevertheless, these structures have been also related to other processes such as coagulation or complement activation, and even with autoimmune pathological processes such as erythematous systemic lupus [6,7,8]. Since its discovery, one of the central issues in the study of NETs has been the understanding of the mechanism associated with its release. Death of neutrophils through NETosis is not a random event; on the contrary, it is a highly regulated process involving a series of sequential steps [9,10,11]. Initially, neutrophils detect pathogens through receptors on cell surface such as TLRs, dectins, integrins or antibody receptors [12,13,14,15]. Then, diverse signaling pathways are activated to drive events to lead NETotic commitment, resulting in reactive oxygen species (ROS) generation, histone processing, disassembling of a nuclear envelope and dissolution of internal membranes [16,17,18,19]. Decondensed DNA is then associated with antimicrobial proteins from cytoplasmic granules such as neutrophil elastase (NE), myeloperoxidase (MPO), cathepsin G (CG), proteinase 3 (PR3) and others [1]. Finally, cytoplasmic membrane ruptures and NETs are released [20].

Multiple mechanisms of NETosis have been identified depending on the stimulus used to trigger the process, including nuclear, mitochondrial or blebbing NETs [1,21,22]. The diverse mechanisms seem to differ from each other in the receptors involved, the signaling pathways activated or the ROS source required.Phorbol 12-myristate 13-acetate (PMA) and calcium ionophores (A23187 and ionomycin) are widely used stimuli to study NETosis; nevertheless, they have the disadvantage that they are stimuli with little biological relevance [23,24]. Despite this, the findings obtained with PMA and calcium ionophores have allowed us to decipher part of the process, mainly the critical need for ROS generation [25,26]. While ROS derived from NADPH oxidase was linked to PMA-induced NETosis [27], NETosis triggered by calcium ionophores does not require NADPH oxidase activity, although that depends partially on mitochondrial ROS (mitROS) [28]. Therefore, NETosis mechanisms have been classified into two groups: NADPH oxidase dependent or independent [29]. NAPDH-independent NETosis is not limited to the generation of mitROS as reactive nitrogen species (RNS), as well as exogenous ROS sources could also serve to trigger NET release [30,31]. In this context, it has been demonstrated that microorganisms such as *Escherichia coli* and *Mycobacterium smegmatis* produce ROS in stress conditions [32]; moreover, ROS from *Candida albicans* are responsible for triggering NET release in neutrophils from patients with chronic granulomatous disease [33].

*Entamoeba histolytica* is the protozoan responsible for causing intestinal (amoebic colitis or dysentery) and extraintestinal (amoebic liver abscess) amoebiasis in humans [34]. This parasite represents a public health problem, especially in developing countries where a prevalence of 1% to 20% in the population and up to 50% of diarrhea due to amoebiasis in infants have been reported [35,36,37]. Immune response activation against amoeba implicates a rapid recruitment of neutrophils to the infection site [38]; however, the exact role of these cells in amoebiasis remains unknown. Thus, some experiments suggest that neutrophils are required for clearance of the infection [39], whereas other evidence suggests that these cells could play a pathological role [40]. We previously showed that *E. histolytica* trophozoites induce a rapid NETosis in human neutrophils that is dependent on the viability of the parasite but independent of NADPH oxidase and PAD4 activities [41]. Nevertheless, a ROS source has not been identified. In this report, we investigated the source of ROS that leads to *E. histolytica* trophozoites-induced NETosis.

## 2. Materials and Methods

### 2.1. E. histolytica Trophozoites

*E. histolytica* trophozoites (HM1:IMSS strain) were axenically cultured in TYI-S-33 medium supplemented with Diamond vitamin tween solution (Merck) and 15% heat-inactivated adult bovine serum (Microlab). Trophozoites were grown for 72 h at 37 °C until they reached the log phase and harvested by chilling on ice for 5 min and centrifugation at 1400 rpm during 5 min at 10 °C. The pellet was resuspended in PBS pH 7.4 and the trophozoites counted in hemocytometer and preserved at room temperature until use. For selected experiments, amoebas (5 × 10^5^) were resuspended in 500 µL of PBS and fixed with formaldehyde (3.7%) or heat-inactivated (56 °C) for 30 min in both cases.

### 2.2. Neutrophil Isolation

Neutrophils were obtained from peripheral blood of healthy volunteers in line with the approach of García–García et al. [42] using Ficoll-PaqueR gradient (GE Healthcare) and hypertonic shock to lyse erythrocytes. Cells were resuspended in PBS pH 7.4, counted in hemocytometer and reserved at 4 °C until use. This study was carried out in accordance with the recommendations and approval of the Ethical Committee for Studies on Humans of the Instituto de Investigaciones Biomédicas, UNAM (Ethical approved number: FMED/CI/RGG/ 013/01/2008). All subjects signed a written informed consent.

### 2.3. NET Quantitation Assay

NET quantitation was performed as described before [43]. In brief, neutrophils (5 × 10^5^) were centrifuged at 4000 rpm for 2 min and resuspended in 500 µL of RPMI-1640 medium (Biological Industries) supplemented with 5% fetal bovine serum (FBS, Gibco) and 500 nM SYTOX^®^ Green (Invitrogen). A volume of 100 µL of cell suspension (1 × 10^5^ neutrophils) was added to a 96 well plate, allowed to sediment for 20 min at 37 °C and then, stimulated with 1 × 10^3^, 2 × 10^3^, 5 × 10^3^ or 1 × 10^4^ *E. histolytica* viable trophozoites (trophozoite:neutrophil ratios 1:100, 1:50, 1:20 and 1:10, respectively). In other experiments, neutrophils were stimulated with 5 × 10^3^ formaldehyde-fixed or heat-inactivated trophozoites. Co-cultures were incubated at 37 °C and fluorescence was measured during 4 h from the well bottom using a spectrofluorometer Synergy HTX (BioTek) with 485 nm excitation and 528 nm emission filters. NETosis induced by PMA (50 nM, Merck) and A23187 (10 µM, Merck) were used as positive controls.

To determine the role of NADPH-oxidase in amoeba-induced NETosis, neutrophils (5 × 10^5^) were resuspended in 500 µL of PBS and pretreated with the inhibitor apocynin (400 µM) or vehicle DMSO (0.1%) for 30 min at 4 °C (all reagents were supplied by Merck). After pretreatment, neutrophils were induced to NETosis with trophozoites as described above. To determine the role of ROS from neutrophils, these cells (5 × 10^5^) were resuspended in 500 µL of PBS and pretreated separately for 30 min at 4 °C with the ROS scavengers pyrocatechol (200 µM), catalase (200 UI/mL), luminol (50, 100 and 200 µM), isoluminol (50, 100 and 200 µM) or mitoTEMPO (400 µM). As negative controls, neutrophils were pretreated with the corresponding vehicles (all reagents were supplied by Merck). After pretreatments, neutrophils were immediately tested for amoeba-induced NETosis as described above in culture media added with the inhibitors or scavengers at concentrations indicated previously (except for mitoTEMPO). All experiments were performed three times in triplicates.

### 2.4. NET Visualization

NET immunofluorescence was performed as described previously [41] with some modifications. In brief, neutrophils (2 × 10^5^) were resuspended in 100 µL of RPMI-1640 medium supplemented with 5% FBS and seeded on coverslips pretreated with poly-L-lysine solution (Merck). After sedimentation for 20 min at room temperature, the neutrophils were stimulated with 1 × 10^4^ viable trophozoites or positive controls PMA (50 nM) and A23187 (10 µM). Co-cultures were incubated for 4 h at 37 °C and then fixed with 3.7% formaldehyde during 10 min. Fixed cells were permeabilized using 0.2% Triton X-100 (BioRad) in PBS for 5 min. Detergent was washed out two times with cold PBS and blocking was carried out with a 1% BSA, 0.3 M glycine and 0.1% Tween 20 in PBS, for 30 min at 37 °C. Samples were then incubated with primary antibodies against NETs constituents: anti-NE (Santa Cruz Biotechnology, sc-365950), anti-MPO (Abcam, ab16886) or anti-acetylated histone H4 (Abcam, ab61238) antibodies diluted 1:100 in 1% BSA, 0.1% Tween 20 in PBS for 1 h at room temperature. Samples were washed two times with cold PBS and then incubated with secondary anti-mouse IgG-FITC (Merck, F5387) or anti-rabbit IgG-TRITC (Zymax) antibodies diluted 1:50 in the same solution as primary antibodies for 1 h at room temperature in the dark. After two washes with cold PBS, samples were stained with 5 µg/mL DAPI (Merck) and the coverslips were mounted on slides using Fluoroshield (Merk) before observation in a fluorescence microscope (Olympus BX51). Images were processed using ImageJ software.

For detection of MPO on the surface of amoebic trophozoites, immunofluorescence was performed as above but cell cultures were fixed for 5 and 10 min after the addition of parasites.

### 2.5. Intracellular ROS Quantitation in Neutrophils

Neutrophils (5 × 10^5^) were resuspended in 500 µL of PBS added with 10 µM 2′,7′-dichlorofluorescein diacetate (H_2_DCFDA) and incubated for 30 min at 37 °C in the dark. Cells were centrifuged at 4000 rpm for 2 min and resuspended in 500 µL of RPMI-1640 supplemented with 5% FBS. Subsequently, each 100 µL of suspension (1 × 10^5^ neutrophils) was transferred to 96 well plate, allowed to sediment for 20 min at 37 °C and then stimulated with 1 × 10^3^, 2 × 10^3^ or 5 × 10^3^ viable *E. histolytica* trophozoites (trophozoite:neutrophil ratios 1:100, 1:50 and 1:20). Fluorescence intensity was measured after incubation during 1 h at 37 °C from the well bottom in the spectrofluorometer Synergy HTX using 485 nm excitation and 528 nm emission filters. PMA (50 nM) and A23187 (10 µM) were used as positive controls for ROS production.

### 2.6. Mitochondrial ROS Quantitation in Neutrophils

Isolated neutrophils (5 × 10^5^) were resuspended in 500 µL of PBS added with MitoSOX^TM^ Red (10 µM) and incubated for 30 min at 4 °C. Cells were centrifuged at 4000 rpm for 2 min and resuspended in 500 µL of RPMI-1640 medium supplemented with 5% FBS. Each 100 µL of MitoSOX pretreated neutrophils (1 × 10^4^) were transferred to 96 well plate and stimulated with 1 × 10^3^, 2 × 10^3^, 5 × 10^3^ or 1 × 10^4^ viable amoebas (trophozoite:neutrophil ratios 1:100, 1:50, 1:20 and 1:10). Fluorescence was read from the well’s bottom after 2 h using a spectrofluorometer Synergy HTX (BioTek) with 485 nm excitation and 580 nm emission filters. PMA (50 nM) and A23187 (10 µM) were used as negative and positive controls of mitochondrial ROS, respectively.

### 2.7. ROS Quantitation in E. histolytica Trophozoites

Viable, formaldehyde-fixed or heat-inactivated trophozoites (5 × 10^5^) were resuspended in 500 µL of PBS added with H_2_DCFDA (100 µM) and incubated for 1 h at 37 °C. After incubation, amoebas were centrifuged at 4000 rpm for 2 min and resuspended in 500 µL of RPMI-1640 medium supplemented with 5% FBS. Each 100 µL of the cell suspension (1 × 10^5^ H_2_DCFDA-pretreated trophozoites) was added to 96 well plate and allowed us to sediment for 10 min at 37 °C. Fluorescence was read from well bottom using a spectrofluorometer Synergy HTX with 485 nm excitation and 528 nm emission filters.

### 2.8. ROS Scavenging from Amoebic Trophozoites

Trophozoites (5 × 10^5^) were resuspended in 500 µL of PBS added with the ROS scavengers pyrocatechol (50, 100 or 200 µM) or luminol (50, 100 or 200 µM), as well as with the vehicle DMSO (0.1%), and incubated during 30 min at 37 °C. Then, the cell suspension was added with H_2_DCFDA (100 µM) and ROS quantitation determined as mentioned above. On the other hand, to determine the role of amoebas-derived ROS in the NETosis process, trophozoites (5 × 10^5^) were resuspended in 500 µL of PBS added with the ROS scavengers pyrocatechol (50, 100 and 200 µM) or luminol (50, 100 and 200 µM). Trophozoites were incubated for 1.5 h at 37 °C, centrifuged at 4000 rpm for 2 min and then resuspended in 500 µL of PBS to be used immediately for NET induction.

### 2.9. Visualization of Amoebas-Derived ROS

Trophozoites treated for ROS quantitation as well as trophozoites treated for ROS depletion with the scavenger pyrocatechol as mentioned above, were fixed with formaldehyde (3.5%) and counterstained with DAPI (5 µg/mL). An aliquot of 20 µL was observed under fluorescence microscope Olympus BX51. Images were processed using ImageJ software.

### 2.10. Detection of MPO Activity

Neutrophils (5 × 10^5^) were centrifuged at 4000 rpm for 2 min and resuspended in 500 µL of RPMI-1640 medium supplemented with 5% FBS (Gibco) and luminol (200 µM) or isoluminol (200 µM). Each 100 µL of cell suspension (1 × 10^5^ neutrophils) was placed in a 96 well plate and cells were incubated for 20 min at 37 °C for sedimentation. Posteriorly, neutrophils were stimulated with 1 × 10^3^, 2 × 10^3^, 5 × 10^3^ or 1 × 10^4^ *E. histolytica* viable trophozoites. Co-cultures were incubated at 37 °C for 4 h and luminescence was measured every 5 min from the well bottom using a spectrofluorometer Synergy HTX. PMA (50 nM, Merck) and A23187 (10 µM, Merck) were used as controls.

### 2.11. Statistical Analysis

Statistical significance was tested with paired two-tailed Student’s *t*-test. Data are reported as mean ± SD. A *p* value ≤ 0.05 was considered statistically significant.

## 3. Results

### 3.1. E. histolytica Trophozoites Induce NETosis in a Dose-Dependent Manner

**Figure 1:**
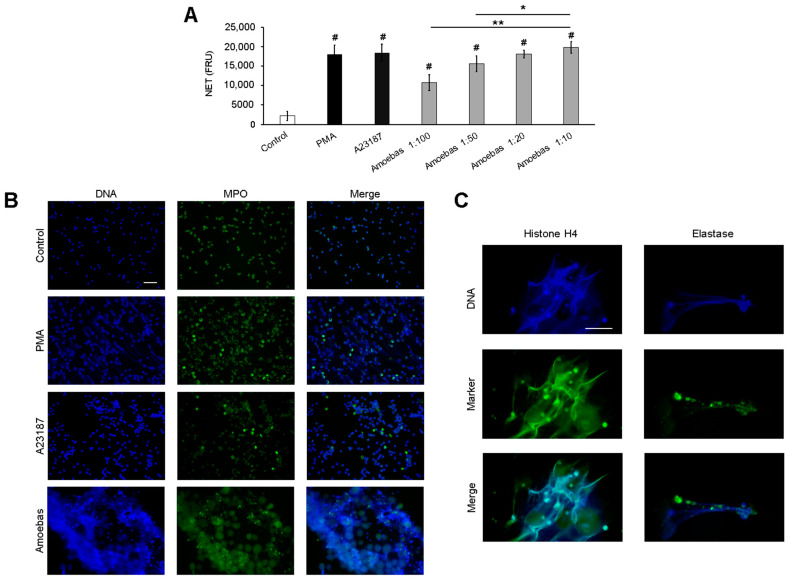
*Entamoeba histolytica* induces NETosis on human neutrophils in a dose-dependent manner. (**A**) Human neutrophils (1 × 10^5^) were cultured with *E. histolytica* trophozoites at ratios amoeba:neutrophil of 1:100, 1:50, 1:20 and 1:10 in RPMI-1640 medium added with 5% FBS and 500 nM SYTOX^®^ Green. PMA (50 nM) and A23187 (10 µM) were used as positive controls of NETosis. Finally, fluorescence was read at 4 h. NETs amount is expressed in fluorescence relative units (FRU). Values are means ± SD of three independent experiments. * *p* < 0.05, ** *p* < 0.001, # *p* < 0.001 respect to control. (**B**) Neutrophils (2 × 10^5^) were stimulated with PMA (50 nM), A23187 (10 µM) or *E. histolytica* trophozoites (1 × 10^4^) during 4 h. After fixation, cells were marked using anti-MPO primary antibody followed by anti-mouse IgG-FITC secondary antibody. DNA was counterstained with DAPI. Images were taken at 40× magnification. Scale bar 100 µm. (**C**) Neutrophils (2 × 10^5^) were co-cultured with 1 × 10^4^ *E. histolytica* trophozoites for 4 h. Cells were fixed and immunofluorescence was performed using anti-NE and or anti-acetylated histone H4 primary antibodies followed by anti-mouse IgG-FITC (for NE) or anti-rabbit-TRITC (for histone) secondary antibodies. DNA was counterstained with DAPI. Images were taken at 100× magnification. Scale bar: 50 µm.

Herein, we evaluate amoeba-induced NETosis under different amoeba:neutrophil ratios. As shown in Figure 1A, amoebas trigger NET release in a dose-dependent manner at ratios between 1:100 to 1:20. The maximum level of DNA release was obtained at ratio 1:20, as no significant differences were observed with respect to the 1:10 ratio and the positive controls PMA and A23187.

NET release was confirmed by detecting MPO in the DNA by immunofluorescence. As shown in Figure 1B, untreated control neutrophils have condensed, multilobed nucleus and MPO located in the cytoplasmic compartment as expected. The positive controls PMA and A23187 induced nuclear decondensation and formation of DNA fibers that were colocalized with MPO. On the other hand, *E. histolytica* trophozoites induced cloudy NETs with heterogenous distribution of MPO. To ensure that these structures correspond to NET, we observed that acetylated histone H4 and NE also colocalized with released DNA in response to trophozoites (Figure 1C).

### 3.2. NETosis Induced by E. histolytica Trophozoites Is Independent of Neutrophil’s ROS

**Figure 2:**
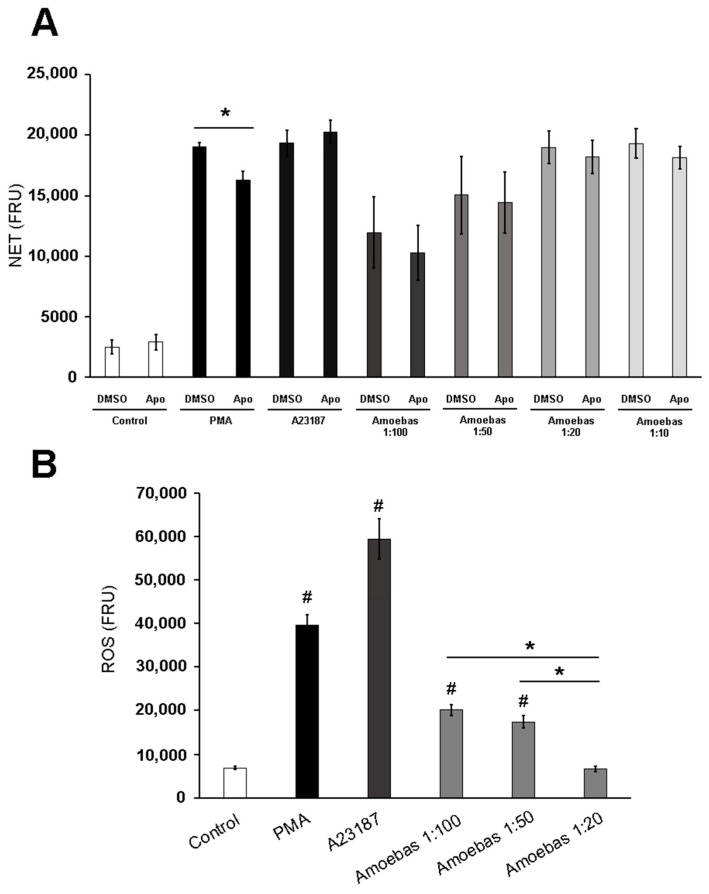
NETosis induced by *E. histolytica* occurs independently of NADPH oxidase. (**A**) Neutrophils (1 × 10^5^) were pretreated with 400 µM apocynin (Apo) or DMSO for 30 min. Posteriorly, cells were transferred to RPMI-1640 medium added with 5% FBS and 500 nM SYTOX^®^ Green and then stimulated with PMA (50 nM), A23187 (10 µM) or *E. histolytica* trophozoites at ratios of 1:100, 1:50, 1:20 and 1:10. Fluorescence was read at 4 h. (**B**) H_2_DCFDA-pretreated neutrophils (1 × 10^5^) were culture in RPMI-1640 medium supplemented with 5% FBS and then stimulated with PMA (50 nM), A23187 (10 µM) or *E. histolytica* trophozoites at ratios 1:100, 1:50 or 1:20. Fluorescence was read at 1 h. NET and ROS amounts are expressed in fluorescence relative units (FRU). Values are means ± SD of three independent experiments. * *p*< 0.0001, # *p* < 0.001 with respect to control.

As shown in Figure 2A, NADPH oxidase inhibitor apocynin significantly reduced NETosis induced by PMA, whereas NETosis induced by A23187 was not affected, as expected. Amoebas-induced NETosis was not affected by apocynin at any trophozoite:neutrophil ratio tested, suggesting that this process is certainly independent of ROS from neutrophils NADPH oxidase (Figure 2A). Then, we evaluated general production of ROS by neutrophils at the different ratios and found that although the respiratory burst was completely suppressed at a 1:20 ratio, as we previously reported, the neutrophils produce ROS at 1:100 and 1:50 ratios (Figure 2B). Taken together, the data strongly suggest that amoeba-induced NETosis is independent of ROS from neutrophils.

### 3.3. Dead Trophozoites Do Not Induce NET Release and Contain Scarce ROS

**Figure 3:**
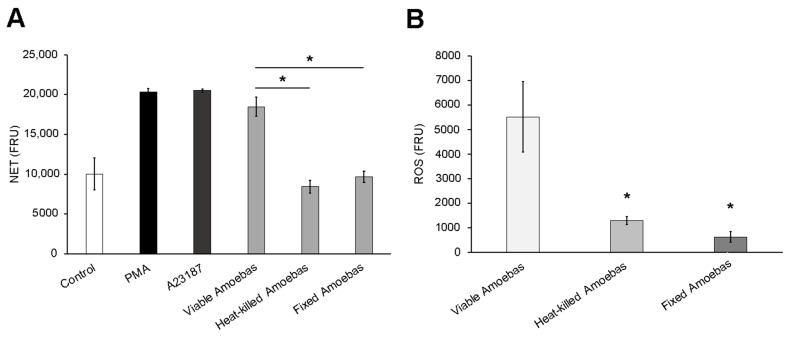
Dead *E. histolytica* trophozoites do not trigger NETosis and possess less ROS. (**A**) Neutrophils (1 × 10^5^) were culture in RPMI-1640 medium supplemented with 5% FBS and added with 500 nM SYTOX^®^ Green. Cells were stimulated with PMA (50 nM), A23187 (10 µM), viable trophozoites (5 × 10^3^), heat-killed trophozoites (5 × 10^3^) or formaldehyde-fixed trophozoites (5 × 10^3^). Fluorescence was read at 4 h. (**B**) Viable, heat-killed and formaldehyde-fixed trophozoites were pretreated with H_2_DCFDA for 1 h. Posteriorly, amoebas (1 × 10^5^) were placed in a 96 well plate and fluorescence was read. NET and ROS amounts are expressed in fluorescence relative units (FRU). Values are means ± SD of three independent experiments. * *p* < 0.0001.

As shown in Figure 3A, heat-killed or fixed trophozoites did not induce NETosis on human neutrophils, in contrast to viable amoebas that induced NET levels like PMA and A23187. This result suggests that metabolically active trophozoites are required for triggering NETosis. Since most studies suggest that NETosis requires a source of ROS, which is not related to neutrophils in this case, we explored the possibility that ROS from trophozoites was involved. So, we decided to quantify ROS in living and dead amoebas and found, as expected, that formaldehyde-fixed and heat-killed trophozoites exhibited far fewer ROS than live amoebas (Figure 3B).

### 3.4. Amoebas Derived ROS Are Required for NETosis

**Figure 4:**
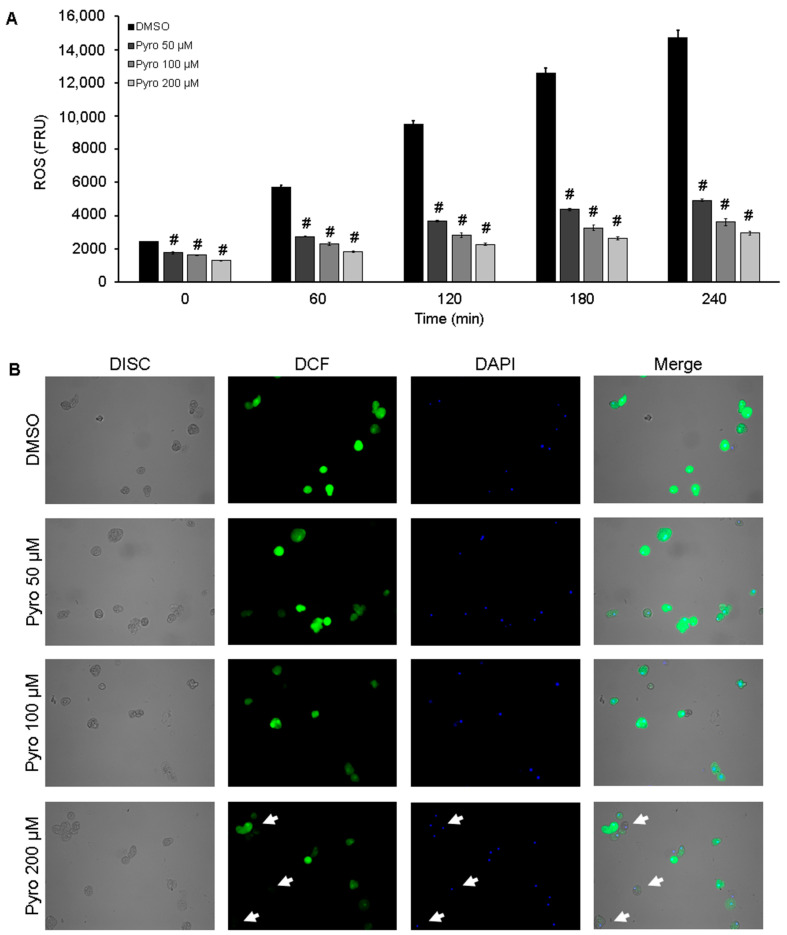
Pyrocatechol reduces ROS generation in viable *E. histolytica* trophozoites. Amoebic trophozoites were treated with DMSO or pyrocatechol (Pyro) at 50, 100 and 200 µM for 30 min and then H_2_DCFDA (100 µM) was added. Cells were incubated for another hour and after treatment, trophozoites were resuspended in RPMI-1640 medium supplemented with 5% FBS. A total of 1 × 10^5^ trophozoites were placed and fluorescence was read every hour during 4 h (**A**) or were fixed and counterstained with DAPI for visualization under fluorescence microscopy (**B**). NET amount is expressed in fluorescence relative units (FRU). Values are means ± SD of three independent experiments. # *p* < 0.01 with respect to the control.

Based on the above results, we evaluated the effect of reducing the amount of ROS in viable trophozoites on their capability to induce NETosis. First, we demonstrated that the pretreatment of *E. histolytica* trophozoites with the ROS scavenger pyrocatechol (50 to 200 µM) for 1.5 h resulted in a significant and dose-dependent reduction of ROS levels. Moreover, this effect persisted during the 4 h period that NETosis assay lasted (Figure 4A). The result was confirmed in H_2_DCFDA-stained trophozoites. As shown in Figure 4B, the amoebic trophozoites exhibited intense green florescence in basal conditions (high ROS levels), whereas the pyrocatechol-treated amoebas exhibited a dose-dependent decrease of green fluorescence denoting a reduction of trophozoite-derived ROS. Many trophozoites pretreated with pyrocatechol 200 µM were virtually non-fluorescent (Figure 4B, lower panel, white arrows). The treatments with pyrocatechol and luminol did not affect the viability of trophozoites (Supplementary Figure S1).

**Figure 5:**
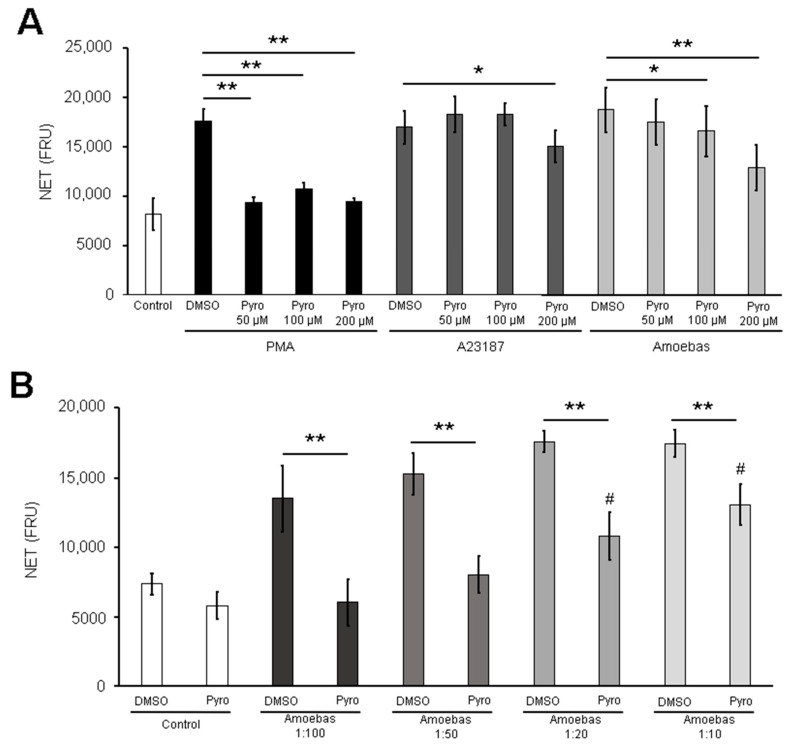
ROS-reduced trophozoites failed to induce NETosis on human neutrophils. (**A**) Neutrophils (1 × 10^5^) were cultured in RPMI-1640 medium supplemented with 5% FBS, 500 nM SYTOX^®^ Green and pyrocatechol (Pyro, 50, 100 or 200 µM or DMSO). Cells were stimulated with PMA (50 nM), A23187 (10 µM) or 5 × 10^3^ pyrocatechol-pretreated trophozoites (according to the concentration present in the medium). Fluorescence was read after 4 h. (**B**) Neutrophils (1 × 10^5^) were cultured in RPMI-1640 medium supplemented with 5% FBS, 500 nM SYTOX^®^ Green and pyrocatechol (200 µM or DMSO). Cells were stimulated with trophozoites pretreated with pyrocatechol (200 µM or the vehicle DMSO) at ratios 1:100, 1:50, 1:20 or 1:10. Fluorescence was read after 4 h. NET amount is expressed in fluorescence relative units (FRU). Values are means ± SD of three independent experiments. * *p* < 0.01, ** *p* < 0.0001, # *p* < 0.05 with respect to the control.

Once we had demonstrated the reduction of ROS in pyrocatechol-pretreated trophozoites, we then carried out NETosis assays with these amoebas. It is noteworthy that ROS-reduced trophozoites induced a reduced NET amount compared with trophozoites pretreated with the vehicle DMSO, and the reduction was statistically significant when pyrocatechol at 100 and 200 µM was used (Figure 5A). As expected, pyrocatechol present in the culture media abolished NET release by PMA at any concentration, but it did not affect NET release by A23187 when used at doses under 100 µM. Surprisingly, A23187-induced NETosis, which is NADPH-ROS independent, was reduced with 200 µM of pyrocatechol (Figure 5A). Involvement of amoeba-derived ROS in NETosis became more evident when lower ratios of parasites per neutrophil were tested. As shown in Figure 5B, trophozoites pretreated with pyrocatechol induced less NET release than DMSO-treated parasites in a dose-dependent manner. Moreover, pretreated amoebas lost the ability to induce NETosis at lower ratios (1:50 and 1:100). It is noteworthy that catalase added to the culture media was unable to prevent NETosis induced by untreated trophozoites (Supplementary Figure S2).

### 3.5. MPO Activity Is Detected Early during Neutrophil-Amoeba Interaction

**Figure 6:**
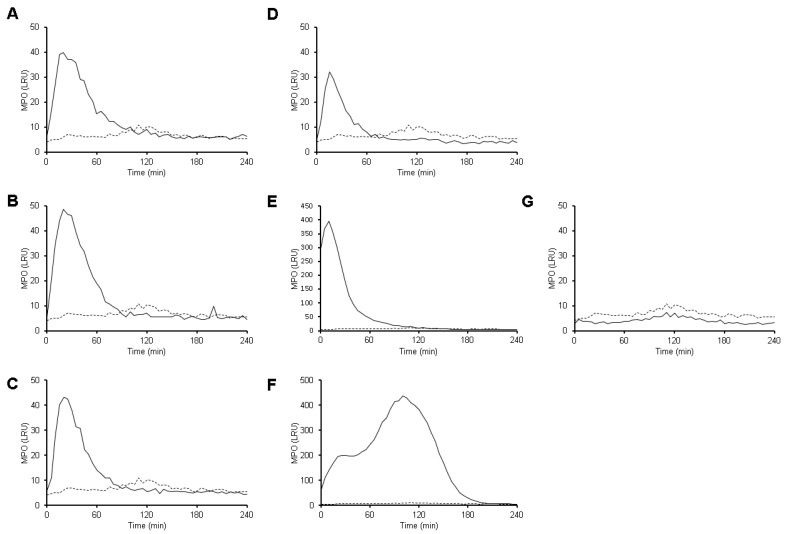
MPO activity is detected during interaction between neutrophils with *E. histolytica*. Neutrophils (1 × 10^5^) were culture in RPMI-1640 medium supplemented with 5%FBS and luminol (200 µM). Cells were stimulated with viable *E. histolytica* trophozoites at ratios of 1:100 (**A**), 1:50 (**B**), 1:20 (**C**) and 1:10 (**D**), as well as 50 nM PMA (**E**), 10 µM A23187 (**F**). Amoebas alone (1 × 10^4^) also were tested (**G**). Black line represents MPO activity (as luminescence relative units, LRU) after stimulation and dotted line represents MPO activity on neutrophils in the absence of stimuli (same for all plots). Values are means of three independent experiments.

Trophozoites induced a rapid MPO activity, detected by luminol, that is independent of the ratio amoeba:neutrophil tested. This activity is detectable in the first minutes of interaction, reaching the maximum value at 20 min (Figure 6A–D). Then, the luminescence signal gradually decreases until it disappears after 90 min. A23187 also induced a similar kinetic of MPO activity that reached the maximum value at 10 min and then, decreased drastically until it disappeared after 100 min (Figure 6E). On the other hand, PMA induced a slower MPO activity that gradually increased during the first 20 min, then reached a steady state for 25 min (Figure 6F). Afterwards, the activity increased and newly reached the maximum value at 100 min, and then decreased to completely disappear at 200 min. It is important to notice that amoebas did not exhibit any MPO activity (Figure 6G).

### 3.6. MPO Activity Is Required for NETosis Induced by E. histolytica

**Figure 7:**
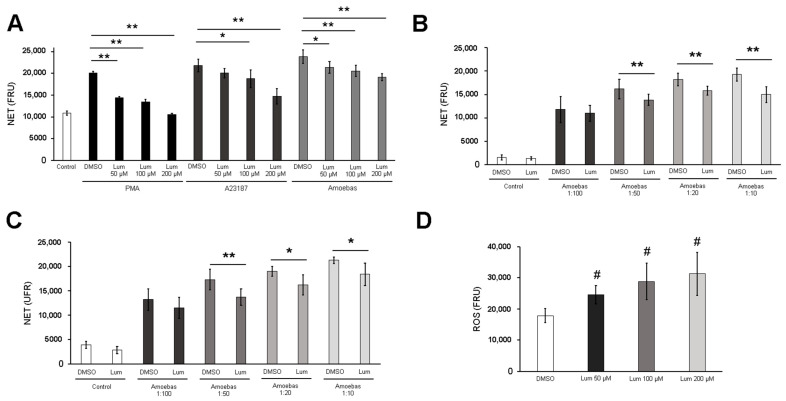
MPO activity is required for NETosis induced by *E. histolytica* trophozoites. (**A**) Neutrophils (1 × 10^5^) were culture in RPMI-1640 medium supplemented with 5% FBS, 500 nM SYTOX^®^ Green and luminol (Lum, 50, 100 or 200 µM or DMSO). Cells were stimulated with PMA (50 nM), A23187 (10µM) or 5 × 10^3^ luminol-pretreated trophozoites (according with concentration present in the medium). Fluorescence was read after 4 h. (**B**) Neutrophils (1 × 10^5^) were pretreated with 50, 100 and 200 µM luminol or DMSO during 30 min. Posteriorly, cells were transferred to RPMI-1640 medium added with 5% FBS and 500 nM SYTOX^®^ Green and then stimulated with PMA (50 nM), A23187 (10 µM) or *E. histolytica* trophozoites at ratios of 1:100, 1:50, 1:20 and 1:10. Fluorescence was read at 4 h. (**C**) Neutrophils (1 × 10^5^) were cultured in RPMI-1640 medium supplemented with 5% FBS, 500 nM SYTOX^®^ Green and luminol (200 µM or DMSO). Cells were stimulated with trophozoites pretreated with luminol (200 µM or the vehicle DMSO) at ratios 1:100, 1:50, 1:20 or 1:10. Fluorescence was read at 4 h. (**D**) Amoebic trophozoites were treated with DMSO or luminol at 50, 100 and 200 µM for 30 min and then H_2_DCFDA (100 µM) was added. Cells were incubated for another hour and after treatment, trophozoites were resuspended in RPMI-1640 medium supplemented with 5% FBS. A total of 1 × 10^5^ trophozoites were placed and fluorescence was read. For (**A**–**D**) the NET or ROS amount are expressed in fluorescence relative units (FRU). Values are means ± SD of three independent experiments. * *p* < 0.01, ** *p* < 0.001, # *p* < 0.0001 with respect to the control.

Luminol, a scavenger of MPO-derived HClO, decreased NET release triggered by *E. histolytica* trophozoites in a dose-dependent manner, suggesting that HClO is involved in this process (Figure 7A). Similar results were observed in PMA and A23187-induced NETosis. When we performed this assay using different trophozoite:neutrophil ratios, luminol (200 µM) decreased NET release induced by trophozoites at 1:50, 1:20 and 1:10 ratios; nevertheless, no differences were observed at a 1:100 ratio (Figure 7B).

To confirm that this effect was due to the reduction of MPO-derived HClO from neutrophils but not a reduction of ROS from amoebas, we pretreated trophozoites with luminol for 1.5 h and then used them to induce NETosis. Surprisingly, a decrease in NET release was observed in a similar way to in the previous experiment (Figure 7C). Therefore, we estimated ROS in luminol-pretreated trophozoites and found that, unlike neutrophils, luminol strikingly caused a dose-dependent increase of ROS in amoebas, as compared to the vehicle DMSO (Figure 7D). Viability of trophozoites was not affected by the treatment with luminol throughout the experiment (Supplementary Figure S1). Taken together, these data suggest that MPO activity from neutrophils and ROS from amoebas is produced early during the contact of the two cells, which is necessary for NETosis.

### 3.7. MPO Is Detected on the Surface of Amoebic Trophozoites Early after Contact with Neutrophils and Its Activity Is Required for Trophozoite-Induced NETosis

**Figure 8:**
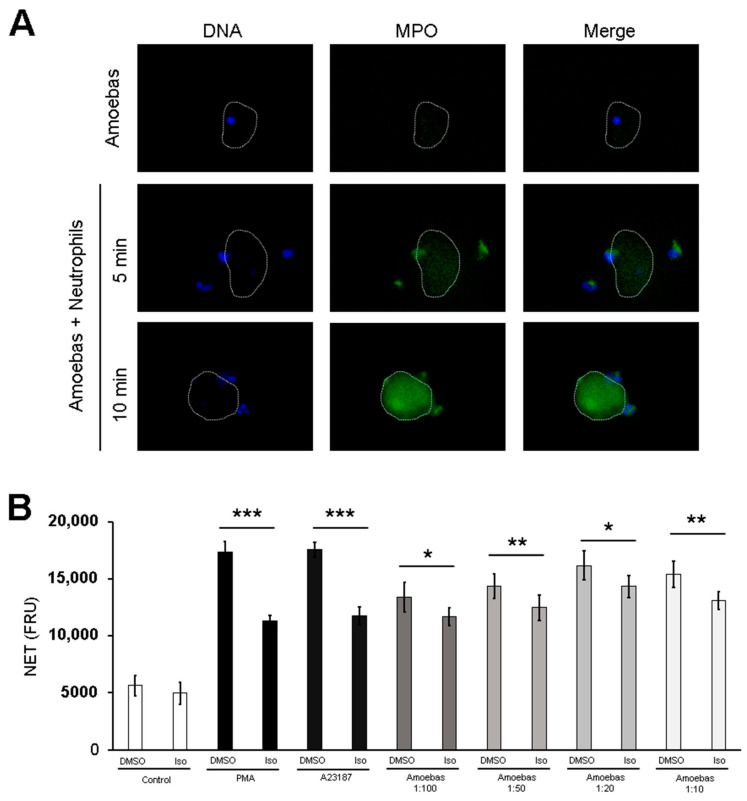
Extracellular MPO activity is required for NETosis induced by *E. histolytica*. (**A**) Neutrophils (2 × 10^5^) were co-cultured with 1 × 10^4^ *E. histolytica* trophozoites during 5 or 10 min. Cells were fixed and immunofluorescence was performed using anti-MPO antibody followed by anti-mouse IgG-FITC secondary antibody. DNA was counterstained with DAPI. Amoebas alone were used as a control. Trophozoites are indicated by doted white lines. Images were taken at 100× magnification. Scale bar 100 µm. (**B**) Neutrophils (1 × 10^5^) were pretreated with 50, 100 and 200 µM isoluminol (Iso) or DMSO for 30 min. Posteriorly, cells were transferred to RPMI-1640 medium added with 5% FBS and 500 nM SYTOX^®^ Green and then stimulated with PMA (50 nM), A23187 (10 µM) or *E. histolytica* trophozoites at ratios of 1:100, 1:50, 1:20 and 1:10. Fluorescence was read after 4 h. NET amount is expressed in fluorescence relative units (FRU). Values are means ± SD of three independent experiments. * *p* < 0.05, ** *p* < 0.01, *** *p* < 0.001.

The above results point out to the possibility of ROS transfer between the two cells in very early stages of contact. As ROS were not detected in neutrophils stimulated with trophozoites at a 1:20 ratio, but since MPO activity was always detected independent of the trophozoite:neutrophil ratio assayed, we explored the possibility that MPO was transferred from the neutrophil to the amoeba. As shown in Figure 8A, anti-MPO antibodies started to react with the surface of trophozoites after 5 min, covering all amoeba surfaces after 10 min of contact with neutrophils, suggesting that MPO was rapidly transferred from neutrophils to amoebas. The fluorescence detected was not due to autofluorescence or unspecific binding of the secondary antibody, as shown in trophozoites alone (Figure 8A).

To determine if the MPO activity in the surface of amoebic trophozoites is involved in NETosis, isoluminol (which does not cross the cell membrane) was used instead of luminol to scavenge MPO-derived HClO. In contrast to luminol, isoluminol reduced NETosis triggered by amoebic trophozoites at all ratios tested, showing significant differences with respect to the control (Figure 8B). Isoluminol also reduced PMA- and A23187-induced NETosis; however, NETosis was not completely abolished.

### 3.8. E. histolytica-Induced NETosis Occurs Independently of Mitochondrial Derived ROS

**Figure 9:**
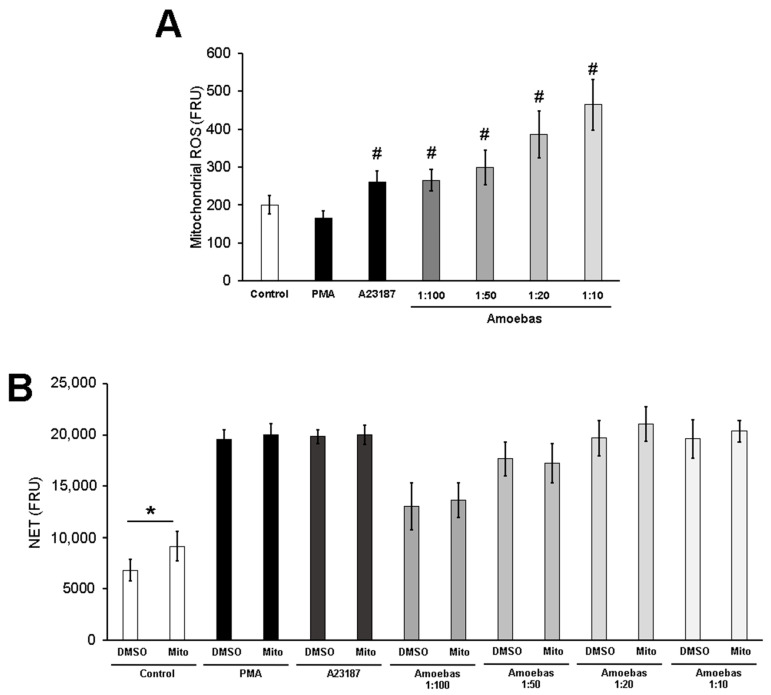
Mitochondrial ROS are not necessary for *E. histolytica*-induced NETosis. (**A**) MitoSOX^TM^ red-pretreated neutrophils (1 × 10^5^) were cultured in RPMI-1640 medium supplemented with 5% FBS and then stimulated with PMA (50 nM), A23187 (10 µM) or *E. histolytica* trophozoites at ratios 1:100, 1:50 or 1:20. Fluorescence was read at 2 h. (**B**) Neutrophils (1 × 10^5^) were pretreated with 400 µM mitoTEMPO (Mito) or DMSO for 30 min. Posteriorly, cells were transferred to RPMI-1640 medium added with 5% FBS and 500 nM SYTOX^®^ Green and then stimulated with PMA (50 nM), A23187 (10 µM) or *E. histolytica* trophozoites at ratios of 1:100, 1:50, 1:20 and 1:10. Fluorescence was read at 4 h. ROS and NET amount are expressed in fluorescence relative units (FRU). Values are means ± SD of three independent experiments. # *p* < 0.001 with respect to the control, * *p* < 0.01.

We decided to explore whether mitochondrial ROS are produced during the NETosis triggered by *E. histolytica* trophozoites. As expected, PMA did not induce mitochondrial ROS, whereas the calcium ionophore A23187 induced a significant increase of these molecules. It is noteworthy that amoebas induced mitochondrial ROS in neutrophils in a dose-dependent manner (Figure 9A). To determine whether mitochondrial ROS are necessary for amoebic-induced NETosis, we used the specific scavenger mitoTEMPO, which did not affect PMA-induced NETosis. As shown in Figure 9B, NETosis was not affected by mitoTEMPO at any ratio tested, suggesting that mitochondrial ROS are not involved.

## 4. Discussion

Neutrophil extracellular traps (NETs) were initially described by Brinkmann et al. [1] as a novel effector mechanism used by neutrophils to entrap and kill bacteria. Since then, many works have explored the mechanism underlying the DNA extrusion to extracellular space, process known as NETosis. Fuchs et al. [27] provided one of the first approaches to understand how NET release takes place, showing that oxidative metabolism directed by NADPH oxidase is involved. Nevertheless, the finding of calcium ionophores triggering NETosis opened the possibility that other mechanisms could also lead to the release of DNA, as they do not require the activity of an NADPH oxidase, but rather require PAD4 activity [44]. The case of NETosis induced by the *E. histolytica* trophozoites is intriguing, as we showed that the process occurs through a non-classical mechanism, independent of NADPH-ROS and PAD4 activity [41,45]. In this work, we performed a set of experiments to continue with the characterization of the amoeba-induced NETosis and found that it is dependent on ROS from *E. histolytica* trophozoites and on the activity of the MPO from neutrophils present in the surface of the parasites.

Previously, we described that amoebic trophozoites triggered NETosis on human neutrophils when co-incubated at trophozoite:neutrophil ratios of 1:20 [41,45,46,47]. Here, we demonstrated that lower amounts of amoebas (ratios 1:100 and 1:50) were also capable of leading NET release in a dose-dependent manner, whereas ratios higher than 1:20 did not induce more NETs. This result indicated that *E. histolytica* trophozoites are one of the most potent parasites to induce NETosis, since other protozoa require greater numbers to trigger significant DNA release. Thus, *Toxoplasma gondii* was used at MOI of 5:1, *Trypanosoma cruzi* at a 1:1 ratio and *Leishmania chagasi*, *L. major* or *L. amazonensis* at 10:1 to 1:1 ratios [12,48,49,50]. In contrast, we observed that 1 amoeba per 100 neutrophils is sufficient to induce NETosis. The reason is unknown but the size of the parasites, and therefore the density of NETosis triggering molecules, could be involved, since *T. gondii* and *Leishmania* forms (amastigotes and promastigotes) do not exceed 15 µm in length and *T. cruzi* trypomastigotes measure 12–30 µm, which is small compared with *E. histolytica* trophozoites measuring up to 60 µm [34,51,52,53]. While neutrophils can phagocyte small parasites enlisted above [54,55,56], they cannot phagocyte amoebas. Instead, we have observed that trophozoites engulf these leukocytes [45]. Neutrophils probably sense the pathogen size through dectin-1, to decide between phagocytosis or NETosis by sequestering of NE [57]. During phagocytosis, NE is moved to the phagolysosome compartment. In contrast, during NETosis, NE is guided to the nucleus for chromatin decondensation. This correlates with our previous observation in which NE is translocated to nuclei during the neutrophil-amoeba interaction [41]. It is conceivable that small parasites mainly drive neutrophils towards phagocytosis, whereas greater pathogens, such as amoebic trophozoites, preferentially induce NETosis.

NETosis mechanisms are generally divided into two groups: dependent on NADPH oxidase activity and independent of NADPH oxidase activity. Fuchs et al. [27] was the first to report that NADPH oxidase inhibition prevented NET release by PMA, and different authors have described the same mechanism for other stimuli [58,59,60]. Later, it was described that calcium ionophores trigger NETosis independently of NADPH oxidase activity, but this mechanism requires PAD4 [44]. We previously reported that *E. histolytica* trophozoites induce NETosis by a non-classical mechanism, independent of NADPH oxidase and PAD4 activities, since apocynin and GSK484, as respective inhibitors, failed to reduce the NET amount. Our previous observations also showed that amoebic trophozoites at 1:20 ratio completely suppressed the oxidative burst in neutrophils [41,46], which has also been reported by others [61]. Interestingly, in this work we found that lower numbers of amoebic trophozoites (1:50 and 1:100 ratios) did not completely abolish ROS generation in these leucocytes. This suggested that neutrophil ROS inhibition by amoebas depends on the density of parasites trespassing a threshold that may cause citrullination of proteins. Accordingly, Zhou et al. [62] showed that dysregulated calcium influx in neutrophils activates PAD4 that citrullinates the cytoplasmatic units p47^phox^ and p67^phox^, blocking the assemble of the NADPH oxidase complex and, in turn, preventing ROS generation. In this context, we previously reported that *E. histolytica* trophozoites trigger calcium influx on human neutrophils and when incubated at a 1:20 ratio [46] and citrullinated proteins were detected [41], which would cause the inactivation of NADPH oxidase. When smaller numbers of trophozoites are confronted, this process may not happen; however, additional studies are required on this. It is worth mentioning that PAD4-independent citrullination of proteins has also been observed during the NETosis induced by *Candida albicans* [63].

Previously, we showed that heat-killed and fixed trophozoites failed to induce NETosis, which was confirmed in this work (Figure 3A) [41]. This data suggests that some products of the *E. histolytica* trophozoites metabolism are responsible for inducing NETosis. In this context, ROS derived from pathogens have been identified as molecules that lead NET release independently of ROS produced by neutrophils [33,43]. Noteworthy, we previously showed that *Entamoeba dispar*, a non-pathogen human amoeba, does not produce ROS and does not trigger NETosis [43,47]. Since most studies suggest that NETosis requires some source of ROS that does not come from neutrophils in this case, we considered the possibility that ROS produced by *E. histolytica* trophozoites induce NETosis. Here we report that viable trophozoites produced basal ROS levels, which agrees with a previous report [64], whereas amoebas killed by heat or fixation with paraformaldehyde produced scarce ROS (Figure 3B). Even though cell death processes have been associated to an increase in ROS production [65], the low level detected by us could be explained by death-inducing agents that were used, since both reduce enzymatic activity and formaldehyde, causing protein cross-linking and the development of heat denaturalizing proteins [66].

When we treated H_2_DCFDA-stained amoebas with hydrogen peroxide, they exhibited a stronger fluorescence (Supplementary Figure S3), suggesting that H_2_DCFDA can be used as an indicator for hydrogen peroxide. Using this approach, we found that pretreatment of amoebas with pyrocatechol, a ROS scavenger of hydrogen peroxide [67,68], reduced the hydrogen peroxide detected in viable amoebas, which is produced as a response to detoxify oxygen through diverse enzymes including NADPH:flavin oxidoreductase (Eh43), thioredoxin reductase (TrxR), NADPH-dependent oxidoreductases (NO1/2) or Fe-superoxide dismutase (FeSOD) [64,69]. The hydrogen peroxide reduction in trophozoites by pretreatment with pyrocatechol impacted the NETosis directly, since a smaller amount of DNA was detected in the extracellular medium. Moreover, NETosis was completely abolished when amoebas were pretreated with pyrocatechol at 200 µM in 1:100 and 1:50 ratios. In accordance with this, pyrocatechol blocked NET release induced by PMA, which is triggered by ROS produced in neutrophils. All these data indicate that amoebic trophozoites, instead of neutrophils, are the source of ROS responsible for leading NETosis involving this parasite. This is the first report regarding the importance of ROS from *E. histolytica* trophozoites for NETosis.

Kenny et al. [33] proposed that hydrogen peroxide produced by *C. albicans* was able to enter to neutrophils to trigger NETosis. In our case, however, the addition of catalase (which possesses high specificity for hydrogen peroxide) [70] to the media in the cocultures amoeba-neutrophil failed to reduce NETosis. In contrast, catalase reduced significantly NET release induced by PMA, which has been linked to extracellular production of hydrogen peroxide by NADPH oxidase in the plasma membrane [27,71]. As amoebic hydrogen peroxide is important for amoeba-induced NETosis but catalase did not affect the process, we proposed that another ROS, probable derived from hydrogen peroxide released by the trophozoites but produced in the extracellular media, might be involved. This ROS may be produced by the activity of a neutrophil product released very early after the contact with amoebas. In this context, neutrophil MPO has been related to some mechanisms of NETosis [72], mainly with non-phagocytosed stimuli [73,74]. This enzyme produces hypochlorous acid (HClO) from hydrogen peroxide and chloride during oxidative burst in neutrophils [75]. Although luminol can react with other oxidants, Gross et al. [76] reported that its luminescence depends substantially on MPO activity. Here we observed that luminol-pretreated trophozoites when incubated with neutrophils exhibit MPO activity denoted by an increase in luminol signal. It is noteworthy that the MPO maximum activity was detected when the DNA extrusion started (approximately 20 min), suggesting that this could be the triggering stimulus. In addition, amoebas did not produce a luminol signal in the absence of neutrophil, indicating that the signal detected in amoebas corresponds to the activity of neutrophil´s MPO and not to other molecules produced by trophozoites or neutrophils. The role of MPO activity on amoeba-induced NETosis was confirmed by taking advantage of the luminol ability to scavenge HClO [77]. Luminol, at all concentrations tested here, reduced NET release induced by the amoebic trophozoites at 1:50, 1:20 and 1:10 ratios. As pretreatment of amoebas with luminol showed similar results but increased ROS levels in the parasite, the data together indicated that NET reduction was due to the scavenging activity of luminol on MPO derived ROS (HClO), instead of a decrease in ROS from amoebas. The reason why luminol increased the ROS of amoeba is unknown. We suspect that luminol can cause intensive stress in the trophozoites without affecting their viability, but this is a subject for further study in our laboratory. It is also interesting that luminol was unable to reduce NETosis at a 1:100 ratio and only reduced but did not abolish NETosis at higher ratios, suggesting that other mechanisms take place to compensate for NET release depending on the culture conditions. In this context, it has been reported that some pathogens such as *L. amazonensis* and *C. glabrata* can trigger NETosis by different mechanisms [50,59].

The role of mitochondrial ROS (mitROS) in amoeba-induced NETosis was also explored. Douda et al. [28] stated that ROS derived from mitochondria are required for the NETosis induced by the calcium ionophores ionomycin and A23187, results that have been replicated [78]. Since then, other NET inducers such as *Leishmania* parasites or UV light have been shown to require mitROS [50,79]. Here we observed for the first time that *E. histolytica* trophozoites induce production of mitROS on human neutrophils in a dose dependent manner. How they are generated is unknown but the recognition of amoebic LPPG by TLR2 and TLR4 [80] and the stimulation of calcium influx plus a pathological stimulus (such as PAAR detected through TLRs) could be involved, as suggested elsewhere [81,82]. Although mitROS were detected in neutrophils in contact with amoebas, the scavenger mitoTEMPO failed to reduce NET releases, suggesting that they do not participate in the process.

Immunofluorescence performed on amoeba-neutrophil cocultures exhibited that DNA from neutrophils is released as aggregated cloudy NETs in accordance with a previous classification [83]. We observed extensive areas covered by DNA that entrap trophozoites (Figure 1B). Differing from other stimuli such as LPS, monosodium urate crystals or S-nitroso-N-acetyl-D,L-penicillamine, [84,85,86], NETs induced by trophozoites do not exhibit an homogeneous distribution of MPO and NE; in contrast, these proteins are usually visualized as spots located in reduced areas, suggesting that at least part of the neutrophil MPO and NE proteins are released by degranulation, and bound to trophozoites before, or at the same time, as NETs [47], which can explain the scarce proteins associated with DNA. Binding of MPO to *E. histolytica* trophozoites has be described previously by Pacheco–Yépez et al. [87]. They reported that purified MPO interacts with amoebic trophozoites, leading to morphological changes and loss of viability, which were also observed by us in amoebas entrapped in NETs [47].

**Figure 10:**
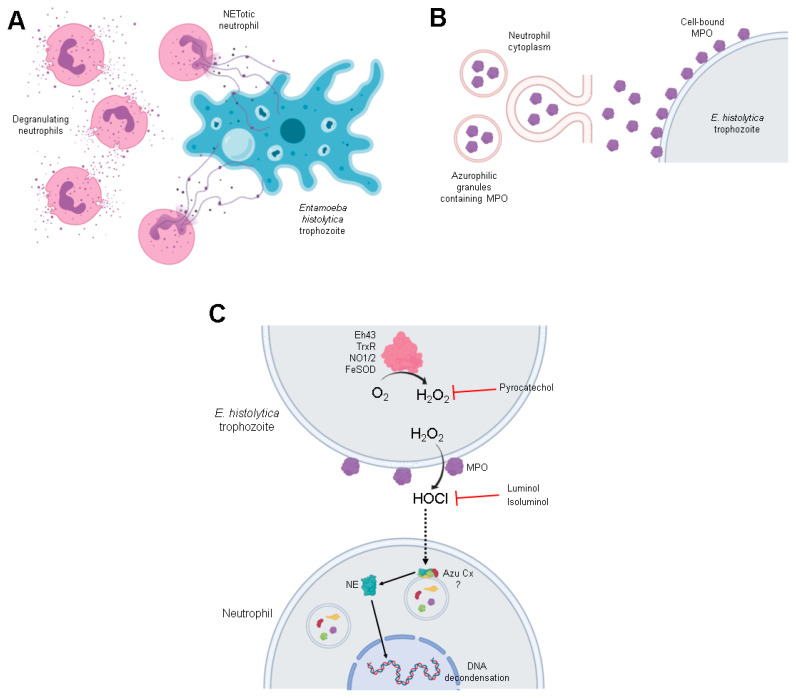
Mechanism proposed for NETosis induced by *E. histolytica*. (**A**) Amoebic trophozoites lead neutrophils to degranulation and NET release. (**B**) MPO derived from azurophilic cytoplasmatic granules is released and it binds to the surface of trophozoites. (**C**) During the oxidative metabolism of *E. histolytica*, H_2_O_2_ is generated to detoxify O_2_ by diverse enzymes such as Eh43, TrxR, NO1/2 or FeSOD. H_2_O_2_ is rapidly converted to HClO through MPO bounded to cell surface of amoebas. HClO probably enters neutrophils by an unknown mechanism and it starts NETosis, promoting NE translocation to the nucleus. Pyrocatechol blocked NETosis scavenge H_2_O_2_ inside amoebas, while luminol and isoluminol react with HClO. Image was made in BioRender.com. Eh43 (NADPH:flavin oxidoreductase), TrxR (thioredoxin reductase), NO1/2 (NADPH-dependent oxidoreductases), FeSOD (Fe-superoxide dismutase), H_2_O_2_ (hydrogen peroxide), HOCl (hypochlorous acid), Azu Cx (azurosome complex), NE (neutrophil elastase).

Finally, detection of MPO covered trophozoites during neutrophil-amoeba interaction raise a question about the origin of this enzymatic activity. Therefore, we decided to use isoluminol, a hydrophobic isomer of luminol, to explore the scavenging of extracellular HClO [88]. Isoluminol significantly reduced NET amount released in response to *E. histolytica* trophozoites at the same level as luminol, indicating that extracellular MPO activity is the responsible for NETosis. This result is in accordance with previous observations indicating that exogen HClO and hypochlorite are sufficient to induce NETosis on human neutrophils [31,89]. Although the role of MPO activity in NETosis has been controversial, with some reports indicating that is dispensable [90,91] and others indicating that is required [73,77], our results support the latter. Taken together, the mechanism that we propose for amoebic-induced NETosis is shown in Figure 10.

## 5. Conclusions

In conclusion, our data show that after detecting each other, neutrophils rapidly transfer MPO to the surface of the amoeba, and that its activity could be processing the ROS of the amoebae that are necessary for the optimal induction of NETosis. In the context of parasite-neutrophil interaction, this observation is very relevant, as many parasites can inhibit respiratory burst in neutrophils but NETosis is easily induced. In general, this contribution supports the notion that parasite-induced NETosis is a very complex process, involving the rapid and active exchange of molecules between parasite and host cells.

## Data Availability

Data is contained within the article and supplementary material.

## Supplementary Materials

The following are available online at https://www.mdpi.com/article/10.3390/antiox10060974/s1, Figure S1: Pyrocatechol and luminol do not affect the viability of *E. histolytica* trophozoites, Figure S2: Catalase failed to reduce NET induced by *E. histolytica*, Figure S3: H_2_DCFDA detects hydrogen peroxide in trophozoites.
