# Supplementary material for: New Insights on NETosis Induced by Entamoeba histolytica: Dependence on ROS from Amoebas and Extracellular MPO Activity
Source: Antioxidants (Basel). 2021 Jun 18;10(6):974. doi: 10.3390/antiox10060974 (PMC8233886; doi:10.3390/antiox10060974)
Supplement: Supplementary file 1 [file antioxidants-10-00974-s001.zip › antioxidants-1241721-supplementary.pdf]

Supplementary material

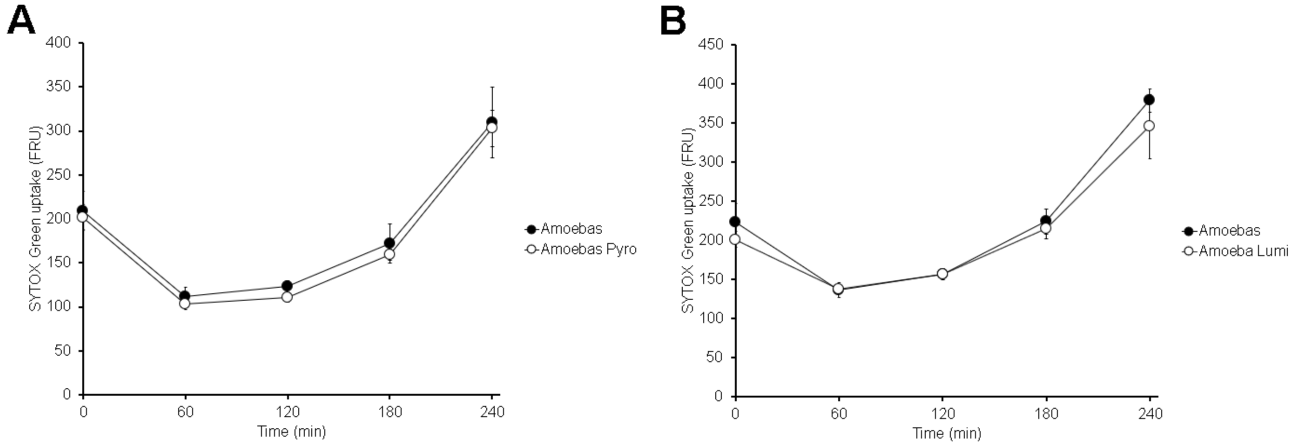

Figure S1: Pyrocatechol and luminol do not affect the viability of *E. histolytica* trophozoites. (A) Amoebas (5×10^5) were pretreated during 1.5 h with pyrocatechol (Pyro, 200 µM) or the vehicle DMSO. Posteriorly an aliquot of 20 µl (1×10^4 trophozoites) were transferred to 100 µl of RPMI-1640 medium supplemented with 5% fetal bovine, 500 nM SYTOX® Green and 200 µM pyrocatechol. Allowed to sediment for 20 min at 37 °C and then fluorescence was measured at 4 h from the well bottom using a spectrofluorometer Synergy HTX with 485 nm excitation and 528 nm emission filters. (B) Amoebas (5×10^5) were pretreated during 30 min with luminol (Lumi, 200 µM) or the vehicle DMSO. Posteriorly an aliquot of 20 µl (1×10^4 trophozoites) were transferred to 100 µl of RPMI-1640 medium supplemented with 5% fetal bovine, 500 nM SYTOX® Green and 200 µM pyrocatechol. Allowed to sediment for 20 min at 37 °C and then fluorescence was measured at 4 h from the well bottom using a spectrofluorometer Synergy HTX with 485 nm excitation and 528 nm emission filters. SYTOX® Green uptake is expressed in fluorescence relative units (FRU). Values are means ± SD of three independent experiments.

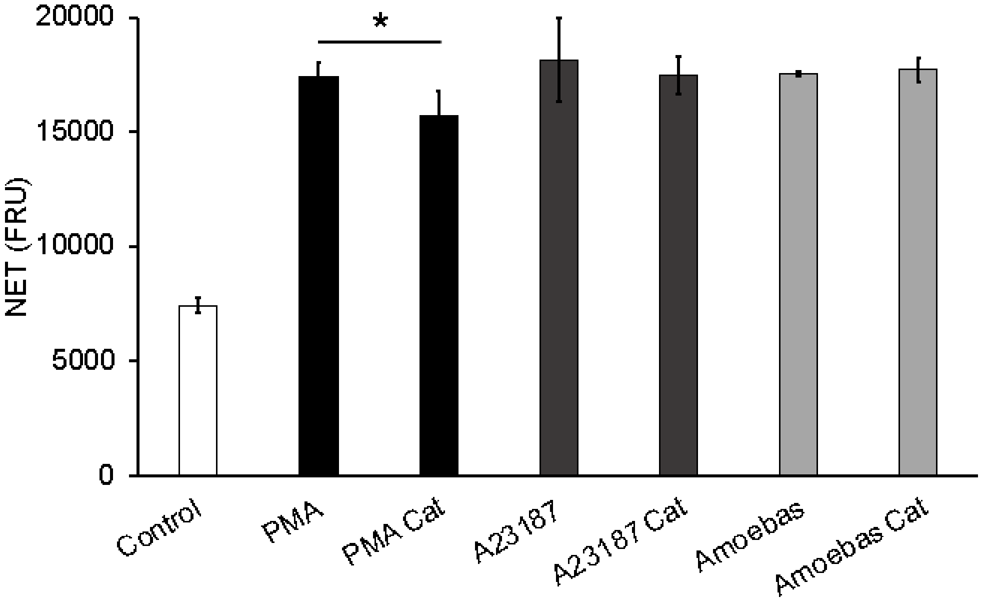

Figure S2: Catalase failed to reduce NET induced *by E. histolytica*. Neutrophils (5 × 10^5) were resuspended in 500 µL of RPMI-1640 medium supplemented with 5% fetal bovine, 500 nM SYTOX® Green and 200 UI/ml catalase (B). A volume of 100 µL of cell suspension (1 × 10^5 neutrophils) was added to a 96 well plate, allowed to sediment for 20 min at 37°C and then, stimulated with 5 × 10^3 *E. histolytica* viable trophozoites. Co-cultures were incubated at 37°C and fluorescence was measured at 4 h from the well bottom using a spectrofluorometer Synergy HTX with 485 nm excitation and 528 nm emission filters. NETosis induced by PMA and A23187 were used as positive controls. NET amount is expressed in fluorescence relative units (FRU). Values are means ± SD of three independent experiments. *p<0.05.

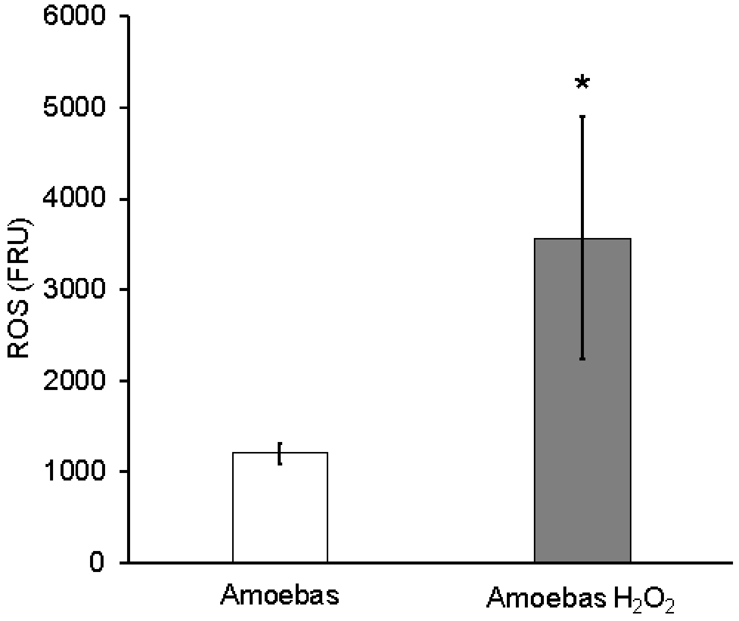

Figure S3: H_2DCFDA detects hydrogen peroxide in trophozoites. *E. histolytica* trophozoites (5×10^5) were resuspended in 500 µL of PBS added with H_2DCFDA (100 µM) and incubated for 1 h at 37°C. After incubation, amoebas were centrifuged at 4,000 rpm for 2 min and resuspended in 500 µL of RPMI-1640 medium supplemented with 5% FBS. Each 100 µL of the cell suspension (1×10^5 H_2DCFDA-pretreated amoebas) was added to 96 well plate and allowed to sediment during 10 min at 37 °C. Hydrogen peroxide (3%) or water was added. Fluorescence was read from well bottom using a spectrofluorometer Synergy HTX with 485 nm excitation and 528 nm emission filters. ROS amount is expressed in fluorescence relative units (FRU). Values are means ± SD of three independent experiments. *$p<0.001$.
